# Conditioned media from macrophages of M1, but not M2 phenotype, inhibit the proliferation of the colon cancer cell lines HT-29 and CACO-2

**DOI:** 10.3892/ijo.2013.2203

**Published:** 2013-12-02

**Authors:** ALEXANDER ENGSTRÖM, ANN ERLANDSSON, DICK DELBRO, JONNY WIJKANDER

**Affiliations:** 1 Department of Health Sciences, Karlstad University, Karlstad;; 2 School of Health and Medical Sciences, Örebro University, Örebro, Sweden

**Keywords:** M1 macrophages, M2 macrophages, THP-1, colon cancer cell line, HT-29, CACO-2

## Abstract

Solid tumors are infiltrated by stroma cells including macrophages and these cells can affect tumor growth, metastasis and angiogenesis. We have investigated the effects of conditioned media (CM) from different macrophages on the proliferation of the colon cancer cell lines HT-29 and CACO-2. CM from THP-1 macrophages and monocyte-derived human macrophages of the M1 phenotype, but not the M2 phenotype, inhibited proliferation of the tumor cells in a dose-dependent manner. Lipopolysaccaharide and interferon γ was used for differentiation of macrophages towards the M1 phenotype and CM were generated both during differentiation (M1_DIFF_) and after differentiation (M1). M1 and M1_DIFF_ CM as well as THP-1 macrophage CM resulted in cell cycle arrest in HT-29 cells with a decrease of cells in S phase and an increase in G_2_/M phase. Treatment of HT-29 cells with M1_DIFF_, but not M1 or THP-1 macrophage CM, resulted in apoptosis of about 20% of the tumor cells and this was accompanied by lack of recovery of cell growth after removal of CM and subsequent culture in fresh media. A protein array was used to identify cytokines released from M1 and M2 macrophages. Among the cytokines released by M1 macrophages, tumor necrosis factor α and CXCL9 were tested by direct addition to HT-29 cells, but neither affected proliferation. Our results indicate that M1 macrophages inhibit colon cancer cell growth and have the potential of contributing to reducing tumor growth *in vivo*.

## Introduction

Colorectal cancer (CRC) is one of the most prevalent cancers and the fourth leading cause of cancer death worldwide (1,2). Approximately 70% of all CRC is sporadic, i.e. non-familial, non-hereditary and unrelated to inflammatory bowel diseases (3,4). The etiology of CRC has not been elucidated, so far, but there are strong indication of the significance of dietary as well as microbiological factors (5,6). In contrast, the pathogenesis of sporadic CRC is well established. Thus, malignant transformation of colorectal epithelial cells is achieved according to the adenoma-carcinoma sequence in which sequential mutations of growth controlling genes, along with epigenetic events occur at a time-course of probably 10–15 years (7,8). Although there is a deep understanding of the genetic basis of CRC, the importance of contributing factors to CRC progression in the tumor stroma is still unclear.

Solid cancers consist of tumor cells that are supported by a scaffold of connective tissue (i.e. the stroma), together with a variety of stromal cells, like fibroblasts, myofibroblasts, endothelial cells, lymphocytes, mast cells and macrophages (9,10). The stroma interacts with the tumor cells, e.g. via cytokines, integrins and proteases, to influence functions such as proliferation, apoptosis, migration and angiogenesis (11–14).

Among the stromal cells, the macrophages are of particular significance for carcinogenesis. Tumor-associated macrophages (TAMs) are experts in changing their functional profiles in response to environmental changes and display a phenotypic plasticity with two main types of macrophages, M1 and M2, with usually contrasting effects on tumor cells (15–18).

M1 macrophages are the classically activated macrophages that respond to signals such as bacterial stimuli with a strong inflammatory response that includes pro-inflammatory cytokines such as interleukin 1β (IL1β), IL6 and tumor necrosis factor α (TNFα), other released factors such as reactive nitrogen/oxygen species and various chemokines that recruits new inflammatory cells to the site (19,20). M2 macrophages are a collection of alternatively activated macrophages that are important in processes such as suppression or regulation of inflammation, wound healing and angiogenesis and release anti-inflammatory cytokines such as IL10 and transforming growth factor β (TGFβ) (21,22).

When human macrophages are exposed to lipopolysaccharides (LPS) and interferon γ (IFNγ), they are polarized to M1 macrophages with potential antitumor activities. When they are exposed to Th2 cytokines, such as IL4 and IL13, they are polarized to M2 macrophages that have been suggested to support tumor growth and development (18,23). TAMs are in most cases regarded as being of an M2 phenotype, but the TAM-picture is probably more complex, and the tumor microenvironment, depending on tissue and cancer type, can affect the polarization of TAMs within the tumor (24–28). The significance of macrophages in CRC is debated since conflicting data regarding extent of macrophage infiltration in correlation to prognosis have been put forward and this may be attributed to differences in macrophage phenotype and localization within the tumor (28–35).

In the current study we have investigated the effect of conditioned media (CM) from human blood monocyte derived M1 and M2 macrophages and THP-1 monocytic cell line derived macrophages on the proliferation of the colon cancer cell lines HT-29 and CACO-2.

## Materials and methods

### Cell culture

The colon cancer cell lines HT-29 and CACO-2 and the acute monocytic leukemia cell line THP-1 were purchased from DSMZ (Braunschweig, Germany). HT-29 cells and THP-1 cells were cultured in RPMI-1640 (RPMI) (Life Technologies, Carlsbad, CA, USA) supplemented with 2 mM L-glutamine (Life Technologies), 100 U/ml penicillin and 100 *μ*g/ml streptomycin (Life Technologies) with 10% heat-inactivated fetal calf serum (FCS) (Thermo Scientific, Waltham, MA, USA) and 10 mM HEPES (Life Technologies). CACO-2 cells were cultured in DMEM supplemented with 2 mM L-glutamine and 100 U/ml of penicillin and streptomycin with 10% FCS. All cell lines were grown at 37°C in a humidified atmosphere and 5% CO_2_.

For the experimental assessment of proliferation, apoptosis and cell cycle, HT-29 cells were seeded at a density of 15,000 cells/cm^2^ in RPMI 10% FCS plus 10 mM HEPES onto tissue culture plates (Greiner Bio-One, Frickenhausen, Germany) and allowed to grow for 3 days. After 3 days, the culture media was replaced with macrophage CM, control media (RPMI 5% FCS) or other factors, and treated, usually for 24 h. CACO-2 cells were seeded at a density of 10,000 cells/cm^2^ and grown for 2 days in DMEM 10% FCS before a 48-h treatment and subsequent assessment of proliferation.

### Differentiation of THP-1 monocytes to macrophages

THP-1 monocytes were seeded at a density of 40,000 cells/cm^2^ onto 6-well cell culture plates (BD Biosciences, Franklin Lakes, NJ, USA) in RPMI 10% FCS plus 10 mM HEPES. Cells were induced to differentiate into macrophages with 160 nM phorbol 12-myristate 13-acetate (Sigma-Aldrich, St. Louis, MO, USA). After 24 h, differentiated cells were thoroughly washed 5 times with RPMI 10% FCS and then cultured 48 h in 3 ml RPMI 10% FCS plus 10 mM HEPES to obtain THP-1 macrophage (THP-1 M) CM. The THP-1 M CM was centrifuged to remove cell debris and stored in aliquots at −20°C.

### Isolation of human monocytes and differentiation to macrophages

Buffy coats from healthy blood donors were obtained from the division of Clinical Immunology and Transfusion Medicine, Uppsala University Hospital (Uppsala, Sweden), and monocytes were isolated by gradient centrifugation using Ficoll paque PLUS (GE Healthcare, Little Chalfont, UK). In short, about 50 ml buffy coat was diluted with an equal volume of PBS containing 3 mM EDTA (PBS/EDTA), carefully loaded on Ficoll-Paque PLUS and centrifuged at 900 × g for 30 min at 20°C. The separated mononuclear fraction was collected and diluted with PBS/EDTA followed by centrifugation at 500 × g for 10 min. The pelleted cells were resuspended in PBS/EDTA and washed four times with PBS/EDTA by repeated centrifugations at 200 × g for 10 min. After washing, the cells were resuspended in 100 ml RPMI without FCS and 2 ml of cell suspension was seeded onto 6-well cell culture plates (BD Biosciences) and allowed to adhere for 1.5 h. Non-adherent cells were removed by three washes with PBS and fresh RPMI 5% FCS was added to the wells and cultured overnight. Macrophages were obtained by culturing monocytes for 6 days in RPMI 20% FCS and 20 ng/ml macrophage colony-stimulating factor (M-CSF) (R&D Systems, Minneapolis, MN, USA). After 6 days of culture (with media and M-CSF renewal at day 3) macrophages were washed with PBS and cultured an additional 48 h in RPMI 5% FCS with either no addition generating M0 macrophages, addition of 100 ng/ml LPS (Sigma-Aldrich) plus 20 ng/ml IFNγ (R&D Systems) for M1 differentiation or 20 ng/ml IL4 (R&D Systems) plus 20 ng/ml IL13 (R&D Systems) for M2 differentiation. CM were collected and named M0, M1/M2 differentiation CM (M1_DIFF_ or M2_DIFF_). The differentiated macrophages were washed twice with PBS and cultured for another 48 h in RPMI 5% FCS (without IFNγ/LPS or IL4/IL13) and CM were collected and named M1 and M2 CM, respectively. The collected media were centrifuged to remove cell debris and stored in aliquots at −20°C.

### Proliferation studies of HT-29 and CACO-2

HT-29 and CACO-2 were cultured as described above in the cell culture section before assessment of cell growth after treatment with CM from macrophages, LPS, IFNγ, TNFα (Sigma-Aldrich) or CXCL9 (Prospec-Tany Technogene, East Brunswick, NJ, USA). Cells were loosened by trypsinization using a 0.05% trypsin, 0.02% EDTA solution (Sigma-Aldrich), mixed with an equal volume of trypan blue and counted in a hemacytometer. For the assessment of recovery of cell growth after treatment, cells were washed and then allowed to grow in RPMI 5% FCS for an additional 48 h prior to counting in a hemacytometer.

### Cytokine detection

Cytokines were analyzed in M1_DIFF_ and M2_DIFF_ CM with the RayBio Human Cytokine Antibody Array 3 (Ray Biotech, Norcoss, GA, USA) according to manufacturer’s instructions. Light intensities were detected by exposure to X-ray film. IL10 and IL12p70 content of M1_DIFF_ and M2_DIFF_ CM was analyzed with ELISA (eBioscience, San Diego, CA, USA) according to manufacturer’s instructions and signals detected at 450 nm (570 nm used as reference) with the Infinite M200 pro plate reader (Tecan, Männedorf, Switzerland).

### Apoptosis measurement

HT-29 cells were seeded as described above in the cell culture section and treated for 24 h with CM. Cells were loosened by trypsinization and pooled with their corresponding cell culture media containing eventual floating cells, centrifuged at 300 × g for 5 min and resuspended in 1% paraformaldehyde in PBS. Cell suspensions were incubated on ice for 45 min. Next, cells were washed twice with 5 ml PBS and resuspended in 450 *μ*l ice-cold PBS prior to cell fixation in 5 ml ice-cold 70% ethanol. Fixed cells were stored at −20°C until apoptosis measurements were done using a terminal deoxynucleotidyl transferase dUTP nick end labeling (TUNEL) kit (Phoenix Flow Systems, San Diego, CA, USA) according to manufacturer’s instructions. Cell apoptosis was analyzed on a FACSCalibur (BD Biosciences) flow cytometer and acquired data analyzed with Cell Quest v.3.3 (BD Biosciences).

### Cell cycle analysis

HT-29 cells were cultured as described above in the cell culture section and treated with conditioned macrophage media for 24 h. In some experiments the cells were washed after treatment and allowed to grow for an additional 24, 48 or 72 h in RPMI 5% FCS prior to cell fixation. At the chosen time point, cells were loosened by trypsinization (see above) and pooled with their corresponding cell culture media containing possible loose cells, centrifuged at 200 × g for 5 min and resuspended in PBS with 1% bovine serum albumin (PBS/BSA). Cells were centrifuged again and resuspended in 450 *μ*l ice-cold PBS/BSA prior to fixation in 5 ml ice-cold 70% ethanol. Cells were stored at −20°C until analysis.

Prior to analysis, Triton X-100 was added to a final concentration of 0.1% and cells incubated 10 min at 6°C. Next, cells were centrifuged at 200 × g for 10 min and resuspended in PBS/BSA. Cells were washed an additional time and then resuspended in PBS/BSA and added 0.1% Triton X-100, 50 *μ*g/ml propidium iodide (Sigma-Aldrich) and 200 *μ*g/ml RNase A (Sigma-Aldrich). Samples were incubated at room temperature for 45 min in the dark prior to analysis on a FACSCalibur (BD Biosciences) flow cytometer. Cell cycle distribution was calculated using the ModFit LT software v.3.1 (Verity Software House, Topsham, ME, USA).

### Immunocytochemistry

Human monocyte-derived macrophages of M0, M1 and M2 phenotype were loosened by trypsinization (see above) after 48 h of differentiation and THP-1 macrophages after 24 h. About 50,000–100,000 cells were spun onto a positively charged microscope glass slide (Thermo Scientific) and analyzed using monoclonal antibodies against CD68 (clone KP1, Dako, Glostrup, Denmark) and CD163 (clone 10D6, Novocastra, Leica microsystems, Newcastle, UK). The epitope retrieval procedure for the commercial antibodies was performed as described by the manufacturer. The immunocytochemistry was performed in a Dako autostainer with the EnVision systems reagents (Dako). After immunostaining, the nuclei were counterstained with Mayer’s haematoxylin, dehydrated and mounted using Tissue-Tek coverslipping film (Sakura Finetek, Torrence, CA, USA). Analysis of CD68 and CD163 was performed on at least three separate macrophage batches from different donors. Manual calculations of the percentage of positively stained cells within an area of 450 × 600 *μ*m with approximately 200–400 cells were performed.

### Statistics

Two-sided Student’s t-test was used for all statistical analysis. Paired Student’s t-test was used for all cell counting experiments comparing treated samples vs. untreated controls. The unpaired t-test was used for statistical analysis of apoptosis and cell cycle experiments comparing treated samples vs. untreated controls. Values are presented as mean ± standard deviation (SD) unless otherwise stated. All experiments with macrophages and CM from macrophages were performed with at least 3 different macrophage batches generated from different donors.

## Results

### Characterization of M1 and M2 macrophage phenotypes and THP-1 macrophages

CM was generated from THP-1 macrophages (denoted THP-1 M) as well as from human blood monocyte derived macrophages. The monocyte derived macrophages were either not further differentiated (denoted M0), differentiated with LPS plus INFγ or IL4 plus IL13 to generate M1 and M2 macrophages, respectively. CM were collected both during the differentiation of macrophages (denoted M1_DIFF_ and M2_DIFF_) and after differentiation (denoted M1 and M2). THP-1 macrophages as well as M0, M1 and M2 macrophages all stained positive for CD68 as determined by immunocytochemistry. Regarding CD163 staining, M0 macrophages showed 80±5% positive cells (n=3 macrophage batches) and M2 macrophages 70±10% (n=7 macrophage batches) while M1 macrophages showed only 5±5% positive cells (n=5 macrophage batches) and THP-1 macrophages were negative. M1 macrophages released IL12 (27±27 pg/ml, n=3 macrophage batches) while M0 and M2 macrophages released no detectable IL12 (less than 2 pg/ml). IL10 was released by both M1 macrophages (2,082±472 pg/ml, n=4 macrophage batches) and M2 macrophages (151±95 pg/ml, n=4 macrophage batches).

### Differential effects of conditioned media from macrophages of different phenotypes on HT-29 and CACO-2 proliferation

**Figure 1.**
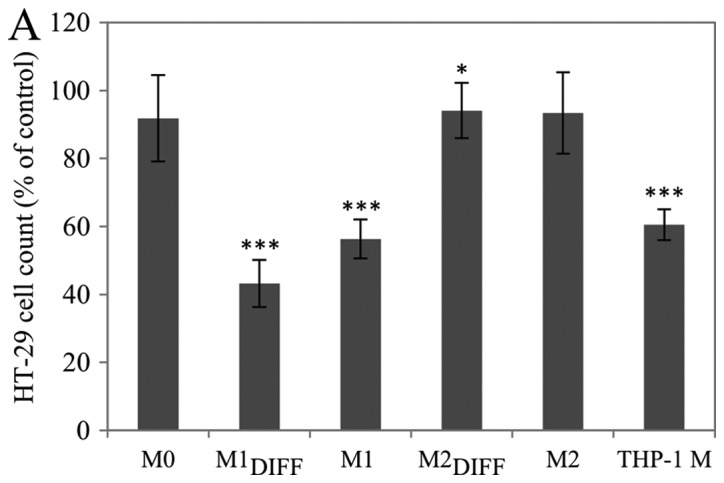

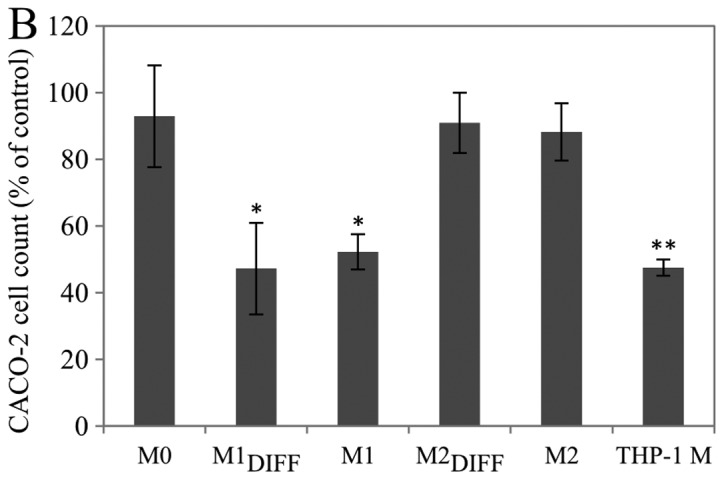

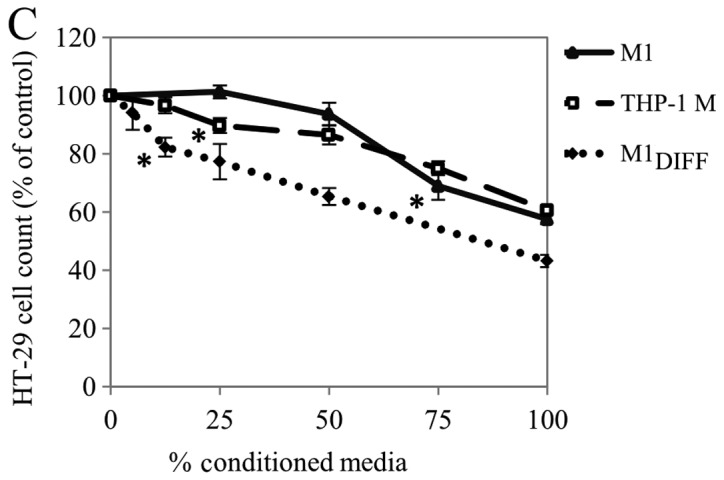
Effect of macrophage CM on the proliferation of HT-29 and CACO-2 cells. (A) HT-29 cells treated 24 h with indicated macrophage CM. (B) CACO-2 cells treated 48 h with indicated macrophage CM. (C) Dose-response of macrophage CM on HT-29 cells treated 24 h. CM was diluted in RPMI 5% FCS to indicated ratios. Cells were counted in a hemocytometer and results are expressed as percent of untreated control (RPMI 5% FCS). Error bars for A, and B, represent SD (A, n ≥9; B, n=4). Error bars for C represent SEM (n ≥4), (^*^denotes the highest dilution of CM that gave a significant reduction in cell number p<0.05). All experiments were performed with at least 4 individual macrophage batches from different donors. ^*^p<0.05, ^**^p<0.01, ^***^p<0.001.

The effect of CM from different macrophage phenotypes on the proliferation of the colon cancer cell lines HT-29 and CACO-2 were investigated. Treatment with either M1_DIFF_ or M1 CM strongly inhibited the proliferation of HT-29 cells, while treatment with M2_DIFF_, M2 or M0 CM had no major effect on the proliferation (Fig. 1A). CM from THP-1 macrophages inhibited proliferation of HT-29 cells by a similar extent as M1 CM. These effects were also seen when the CACO-2 colon cancer cell line was investigated (Fig. 1B). The inhibition of HT-29 proliferation in response to M1_DIFF_, M1 and THP-1 M CM was dose-dependent with M1_DIFF_ CM being the most potent with a significant (p<0.05) inhibition of proliferation already at 1/8th of full dose (Fig. 1C).

In addition to the proliferative inhibition, treatment with M1_DIFF_, but not M1 or THP-1 M CM, also resulted in detachment of HT-29 cells from the culture dishes. The detachment of HT-29 cells varied using different batches of M1_DIFF_ CM and amounted to 17±14% (n=6 macrophage batches) of the total number of cells when treated with full dose of CM.

Control experiments on HT-29 cells with LPS and IFNγ were performed in order to ascertain that the effect of M1_DIFF_ CM was an effect of macrophage released substances and not of the presence of residual LPS/IFNγ. Treatment of HT-29 cells for 24 h with either LPS (100 ng/ml) or INFγ (20 ng/ml) did not suppress proliferation (results not shown). However, the combined treatment with both LPS and INFγ caused a slight reduction in cell numbers compared to control (92.5±7.6%, p<0.05, n=7) and also to some extent generated detachment of cells that amounted to 3±1% of the total number of cells.

M1_DIFF_ CM was more potent than M1 CM regarding inhibition of HT-29 cell proliferation and this was most obvious using 1/2 and 1/4 doses (Fig. 1C). To assess if the more potent anti-proliferative effect of M1_DIFF_ was a synergistic effect between M1 released products and exogenous LPS/IFNγ we added 50 ng/ml LPS + 10 ng/ml IFNγ to 1/2 dose of M1 CM to evaluate if this would increase the potency of M1 CM to that of M1_DIFF_. The addition of LPS plus IFNγ to 1/2 dose of M1 CM only caused a minor (not significant) decrease in cell numbers (81.5±1.5%, n=3) compared to 1/2 dose M1 CM alone (90.5±9.8%, n=3).

**Figure 2.**
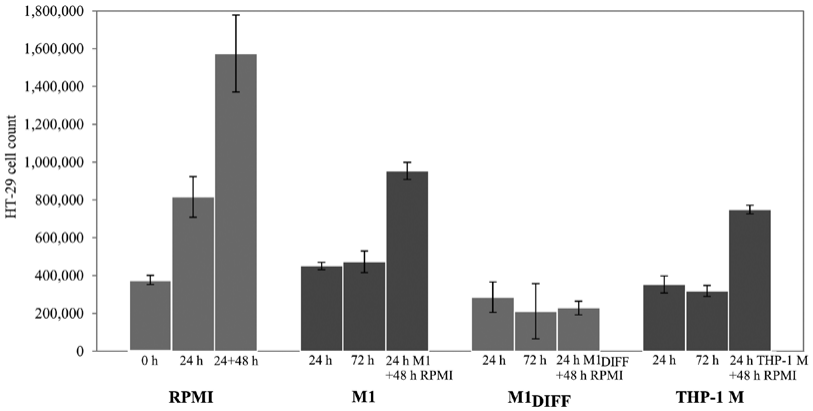
Cell growth recovery of HT-29 cells treated 24 h with macrophage CM. After 24 h CM was replaced with RPMI 5% FCS and cells were allowed to grow an additional 48 h before assessment of cell numbers in a hemocytometer. Twenty-four and 72-h treatments with CM were performed as controls. Viable, adherent cells were counted. Results are mean value ± SD. (RPMI; n ≥5, M1 and M1_DIFF_; n=5, from 4 individual macrophage batches from different donors, THP-1 M; n=3).

To further evaluate the inhibitory effects of M1_DIFF_, M1 and THP-1 M CM on HT-29 cell proliferation we treated cells for 24 h and allowed the cells to recover by further culturing in RPMI 5% FCS for 48 h. While the HT-29 cells that had been treated with M1_DIFF_ CM did not regain their proliferative ability during the 48 h recovery phase, HT-29 cells treated with M1 or THP-1 M CM did so (Fig. 2). Results in Fig. 2 also show an almost complete inhibition of cell growth in cells treated with M1, M1_DIFF_ or THP-1 M CM based on similar cell numbers after treatment compared to cell numbers at start of experiment (compare 0 h with 24 h treatment).

### Conditioned media from different macrophage phenotypes affects apoptosis of HT-29 cells differently

**Figure 3.**
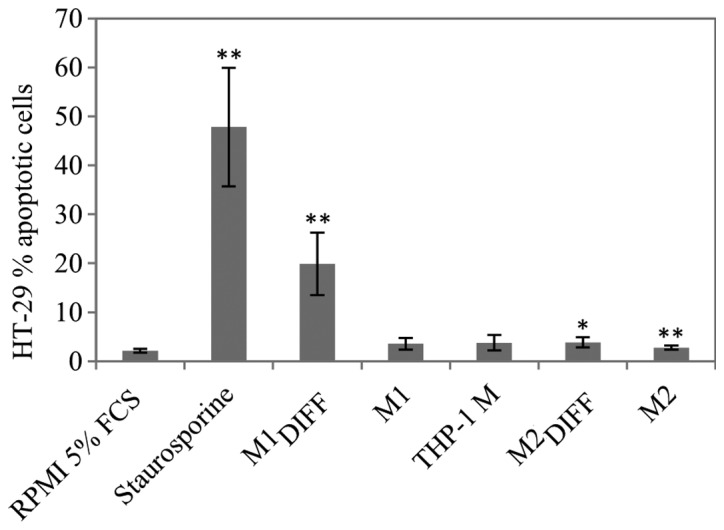
Apoptosis of HT-29 cells treated 24 h with macrophage CM. Analysis was performed with a TUNEL kit and signals detected by flow cytometry. Staurosporine was used as a positive control (1 *μ*M, 24 h). Adherent and floating cells were included in all samples. Results are mean value ± SD from 4 individual batches from different donors. ^*^p<0.05, ^**^p<0.01.

Since there was a decrease in cell count after treatment of HT-29 cells with M1_DIFF_, M1 and THP-1 M CM, apoptosis was determined using a TUNEL assay. M1_DIFF_ was the only CM that induced a major increase of apoptosis in HT-29 cells (Fig. 3). Treatment of HT-29 cells with 1/2 dose of M1_DIFF_ CM also induced apoptosis and to the same extent as full dose (results not shown). Since treatment with M1_DIFF_ CM resulted in detachment of HT-29 cells we also measured apoptosis of detached and adherent cells separately. Almost all of the detached cells gave an apoptotic signal whereas the adherent cells only showed a slight non-significant increase in apoptosis compared to control cells (results not shown).

### Treatment of HT-29 cells with conditioned media from different macrophage phenotypes affects cell cycle distribution

We analyzed if the growth inhibition of HT-29 cells could be explained by cell cycle arrest. After treatment of HT-29 cells with M1_DIFF_, M1 or THP-1 M CM the percentage of HT-29 cells in S phase significantly decreased while the percentage of cells in G_2_/M phase significantly increased indicating a G_0_/G_1_ as well as a G_2_/M cell cycle arrest (Table I). Cell cycle analysis of HT-29 cells that had been allowed to recover in fresh media after treatment with M1 or THP-1 M CM revealed cell cycle distribution similar to control. In contrast, HT-29 cells that had been allowed to recover after treatment with M1_DIFF_ CM showed an accumulation of cells in S phase with very few cells in G_2_/M phase (Table I). In agreement with apoptosis results (Fig. 3) HT-29 cells treated with M1_DIFF_ CM also showed an increase of cells with a sub-G_1_ signal (Table I), another estimate of apoptosis.

### Cytokine and chemokine expression profiles of M1 and M2 macrophages

**Figure 4.**
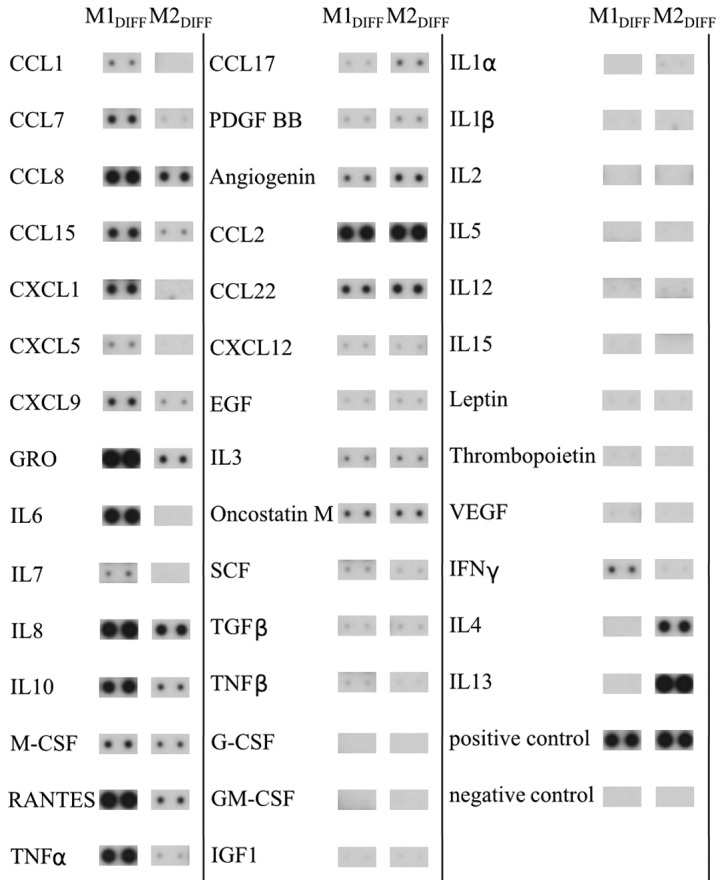
Semi-quantitative expression of 42 different cytokines in M1_DIFF_ and M2_DIFF_ CM analyzed with the RayBio Human Cytokine Antibody Array 3. The shown result is one representative array from n=5 for M1_DIFF_ and n=4 for M2_DIFF_ CM of different macrophage batches from different donors. IFNγ plus LPS were added to M1_DIFF_ and IL4 plus IL13 were added to M2DIFF at start of macrophage differentiation to M1 and M2 macrophages.

In an attempt to identify cytokines and chemokines that could be responsible for the inhibition of proliferation of the colon cancer cell lines we analyzed CM from M1 and M2 macrophages using a protein array. The array demonstrated that M1 macrophages released the cytokines/chemokines TNFα, IL6, IL8, IL10, CCL7, CCL8, CCL15, CXCL1, CXCL9 and RANTES to a much larger extent compared to M2 macrophages (Fig. 4). M2 macrophages released more CCL17 than M1 macrophages. The THP-1 macrophages released a cytokine/chemokine pattern similar to M1 macrophages (results not shown). Among the cytokines detected in M1_DIFF_ CM, TNFα and CXCL9 were added directly to HT-29 cells for evaluation of effects on cell growth. A 24-h treatment with TNFα (100 ng/ml) or CXCL9 (100 ng/ml) to HT-29 gave 104±21% (n=4) and 109±14% (n=5) cells compared to control, respectively.

## Discussion

Macrophages are functionally plastic cells that can adopt two main types, classically activated M1 and alternatively activated M2 phenotypes (17). The existence of macrophages in tumor tissue is well established and there they may influence various aspects of cancer progression including proliferation of tumor cells. In this study we investigated the effect of CM from different phenotypes of macrophages on the growth of the colon cancer cell lines HT-29 and CACO-2. While CM from M0 and M2 macrophages had no effect on the growth of HT-29 and CACO-2 cells a substantial inhibition of the growth was seen in response to CM from THP-1 macrophages and M1 macrophages. The reduction in cell number of the cancer cell lines down to about 50% of control, indicate an almost complete inhibition of growth. The M1_DIFF_ CM was the most potent and a significant inhibition of HT-29 cell growth could be seen using 1/8th of full dose. The M1_DIFF_ CM was generated during the differentiation of the macrophages to M1 and therefore contains residual LPS and IFNγ with the potential of affecting growth of HT-29 cells. A direct addition of LPS plus IFNγ to HT-29 cells, as well as addition of LPS plus IFNγ to a suboptimal dose (1/2 dose) of M1 CM, gave only a minor inhibition of the growth of HT-29 cells. This minor effect by LPS plus IFNγ is therefore less likely to be the sole explanation for M1_DIFF_ CM being substantially more potent than M1 CM. A more plausible explanation is a difference in concentration of the soluble factor/factors present in these two different CM.

The inhibition of HT-29 cell growth in response to THP-1 M, M1_DIFF_ and M1 CM was accompanied by a change in the cell cycle distribution with a decrease of cells in the S phase and increase in the G_2_/M phase, indicating arrest in both G_0_/G_1_ and G_2_/M phases. In addition to the cell cycle arrest, M1_DIFF_ CM, but no other CM induced apoptosis of the HT-29 cells. Furthermore, HT-29 cells that had been treated with M1_DIFF_ CM neither regained proliferation or normalized their cell cycle distribution upon subsequent culture in fresh media which is in agreement with the cells being apoptotic.

Reduced proliferation in response to CM from M1 macrophages has previously been shown for renal clear cell carcinoma (36) and breast cancer cell lines (19). An increase in proliferation has been seen for the colon cancer cell line HCT116 in response to CM from LPS treated murine macrophages of the cell line RAW 264.7 (37). This is in contrast to our results in HT-29 and CACO-2 cells, and also somewhat surprising since the LPS treated RAW 264.7 cells were shown to release substantial amounts of TNFα, IL1 and IL6 indicating a pro-inflammatory phenotype of the macrophages. THP-1 macrophages differentiated with phorbol 12-myristate 13-acetate has been shown to release pro-inflammatory cytokines such as TNFα, IL8 and IL1β (38) and our results reveals similar effects on cancer cell growth and cell cycle regulation between our THP-1 M and M1 CM suggesting an M1-like type of these macrophages.

The macrophages used in our study were characterized by immunocytochemistry and M2 macrophages showed high expression of CD163 while M1 had a low expression, which are results supported by other studies (19,39). The M1 macrophages released IL12 which is in agreement with the general view of this macrophage being of a pro-inflammatory (M1) phenotype (40). Although release of high amounts of IL10 is considered as a hallmark of the M2 phenotype of macrophages (41) we found that both M1 and M2 macrophages released IL10 and that the release was higher from M1 than M2 macrophages. The high release of IL10 from M1 macrophages in our experiments could be explained by the fact that LPS is known to induce IL10 expression in human macrophages differentiated with M-CSF (42).

The cytokine array revealed higher expression of TNFα, IL6, IL8, IL10, CCL7, CCL8, CCL15, CXCL1, CXCL9 and RANTES in M1 compared to M2 macrophages. TNFα is a well known pro-inflammatory cytokine and has in most cases been linked to reduced proliferation of cancer cell lines. In HCT116 cells, a colon cancer cell line, TNFα has been shown to inhibit proliferation (43) and induce apoptosis (44) while in the breast cancer cell line T47D both inhibition (19) and stimulation (45) of proliferation has been seen. We have not been able to observe any effect on the HT-29 cell proliferation in response to a direct addition of TNFα.

CXCL9 is a chemokine that is released in response to IFNγ stimulation of mononuclear cells including macrophages (46). Although less well studied, CXCL9 is thought to be involved in T cell trafficking and has been defined as an anti-angiogenic chemokine (47). Regarding effects on cell growth, CXCL9 has been shown to inhibit intestinal cell proliferation (48) and also to have antitumor activity in a murine cancer model (49). However, we have not been able to see any effects on the HT29 cell proliferation in response to direct addition of CXCL9.

In summary, our results show that CM from THP-1 macrophages and human macrophages of M1 but not M2 phenotype inhibited the growth of the colon cancer cell lines HT29 and CACO-2 and that this was accompanied by cell cycle arrest in G_0_/G_1_ and G_2_/M. Among the cytokines/chemokines selectively released by M1 macrophages, TNFα and CXCL9 did not have any effect on HT-29 cell proliferation suggesting that other factor/factors released by macrophages are responsible for the reduced proliferation and further experiments have to be performed to identify these. Our results imply that the presence of macrophages of the M1 phenotype in the tumor environment would be beneficial for reducing colon cancer cell growth.

## Figures and Tables

**Table I.** Cell cycle analysis of HT-29 cells treated 24 h with macrophage CM with or without a subsequent recovery time in RPMI 5% FCS.

Conditioned medium 24 h treatment	Recovery time in RPMI 5% FCS (h)	G_0_/G_1_ (%)	S (%)	G_2_/M (%)	Sub-G_1_/apoptosis (% of all events)
RPMI 5% FCS	-	67.5±3.9	19.9±3.0	12.6±2.4	2.3±0.7
M1	-	69.4±4.3	4.8±1.3^c^	25.8±4.2^c^	2.8±0.4
M1_DIFF_	-	73.3±2.4^a^	7.1±1.4^c^	19.6±3.2^b^	26.1±12.5^b^
M2	-	73.2±2.7^a^	17.0±5.4	9.7±3.0^a^	2.5±0.4
M2_DIFF_	-	75.1±4.5^a^	16.5±3.6	8.4±3.7^a^	2.7±0.5
THP-1 M	-	76.5±5.7^c^	3.3±0.6^c^	20.3±5.2^b^	2.1±0.3
M1	24	63.1±4.9	22.6±3.7	14.3±1.6	4.3±1.4
THP-1 M	24	63.8±2.5	21.9±2.0	14.3±1.2	3.8±0.8
M1_DIFF_	24	62.3±9.8	36.9±1.4	0.8±1.4	15.4±4.0
M1_DIFF_	48	59.2±7.1	36.8±5.4	4.0±2.1	21.3±8.1
M1_DIFF_	72	72.7±8.2	26.8±8.8	0.6±0.7	28.2±3.6

The sub-G_1_ column shows the percentage of all cells that gave a signal lower than the G_1_ peak and can be used as an estimate of apoptosis. Cell cycle percentages exclude cells that falls outside the G_1_–G_2_ range. Results are mean value ± SD (n ≥6 for all conditions without recovery, n=3 for all conditions with recovery times). Statistical analysis was not calculated for cells with recovery time. n represents an individual experiment and at least 3 different macrophage batches from different donors were used.

a p<0.05,

b p<0.01,

c p<0.001.

## Abbreviations:

CRC: colorectal cancer;
IL: interleukin;
TNF: tumor necrosis factor;
TAM: tumor associated macrophage;
LPS: lipopolysaccharide;
IFN: interferon;
THP-1 M: THP-1 macrophage;
M-CSF: macrophage colony-stimulating factor;
M1: macrophage type 1;
M1_DIFF_: macrophage type 1 during differentiation;
M2: macrophage type 2;
M2_DIFF_: macrophage type 2 during differentiation;
CM: conditioned media

## References

[1] Jemal A, Siegel R, Ward E, Hao Y, Xu J, Thun MJ (2009). Cancer statistics, 2009. CA Cancer J Clin.

[2] Lozano R, Naghavi M, Foreman K (2012). Global and regional mortality from 235 causes of death for 20 age groups in 1990 and 2010: a systematic analysis for the Global Burden of Disease Study 2010. Lancet.

[3] Power DG, Gloglowski E, Lipkin SM (2010). Clinical genetics of hereditary colorectal cancer. Hematol Oncol Clin North Am.

[4] Peyrin-Biroulet L, Lepage C, Jooste V, Gueant JL, Faivre J, Bouvier AM (2012). Colorectal cancer in inflammatory bowel diseases: a population-based study (1976–2008). Inflamm Bowel Dis.

[5] Vargas AJ, Thompson PA (2012). Diet and nutrient factors in colorectal cancer risk. Nutr Clin Pract.

[6] Vipperla K, O’Keefe SJ (2012). The microbiota and its metabolites in colonic mucosal health and cancer risk. Nutr Clin Pract.

[7] Al-Sohaily S, Biankin A, Leong R, Kohonen-Corish M, Warusavitarne J (2012). Molecular pathways in colorectal cancer. J Gastroenterol Hepatol.

[8] Cui G, Shi Y, Cui J, Tang F, Florholmen J (2012). Immune micro-environmental shift along human colorectal adenoma-carcinoma sequence: is it relevant to tumor development, biomarkers and biotherapeutic targets?. Scand J Gastroenterol.

[9] Taketo MM (2012). Roles of stromal microenvironment in colon cancer progression. J Biochem.

[10] Quante M, Varga J, Wang TC, Greten FR (2013). The gastrointestinal tumor microenvironment. Gastroenterology.

[11] Chen JJ, Yao PL, Yuan A (2003). Up-regulation of tumor inter-leukin-8 expression by infiltrating macrophages: its correlation with tumor angiogenesis and patient survival in non-small cell lung cancer. Clin Cancer Res.

[12] Lewis CE, Pollard JW (2006). Distinct role of macrophages in different tumor microenvironments. Cancer Res.

[13] Solinas G, Schiarea S, Liguori M (2010). Tumor-conditioned macrophages secrete migration-stimulating factor: a new marker for M2-polarization, influencing tumor cell motility. J Immunol.

[14] Hanahan D, Weinberg RA (2011). Hallmarks of cancer: the next generation. Cell.

[15] Martinez FO, Gordon S, Locati M, Mantovani A (2006). Transcriptional profiling of the human monocyte-to-macrophage differentiation and polarization: new molecules and patterns of gene expression. J Immunol.

[16] Watkins SK, Egilmez NK, Suttles J, Stout RD (2007). IL-12 rapidly alters the functional profile of tumor-associated and tumor-infiltrating macrophages in vitro and in vivo. J Immunol.

[17] Sica A, Mantovani A (2012). Macrophage plasticity and polarization: in vivo veritas. J Clin Invest.

[18] Biswas SK, Allavena P, Mantovani A (2013). Tumor-associated macrophages: functional diversity, clinical significance, and open questions. Semin Immunopathol.

[19] Rey-Giraud F, Hafner M, Ries CH (2012). In vitro generation of monocyte-derived macrophages under serum-free conditions improves their tumor promoting functions. PLoS One.

[20] Diaz-Gandarilla JA, Osorio-Trujillo C, Hernandez-Ramirez VI, Talamas-Rohana P (2013). PPAR activation induces M1 macrophage polarization via cPLA(2)-COX-2 inhibition, activating ROS production against *Leishmania mexicana*. Biomed Res Int.

[21] Mosser DM, Edwards JP (2008). Exploring the full spectrum of macrophage activation. Nat Rev Immunol.

[22] Pello OM, De Pizzol M, Mirolo M (2012). Role of c-MYC in alternative activation of human macrophages and tumor-associated macrophage biology. Blood.

[23] Ruffell B, Affara NI, Coussens LM (2012). Differential macrophage programming in the tumor microenvironment. Trends Immunol.

[24] Mantovani A, Sica A, Sozzani S, Allavena P, Vecchi A, Locati M (2004). The chemokine system in diverse forms of macrophage activation and polarization. Trends Immunol.

[25] Kim S, Takahashi H, Lin WW (2009). Carcinoma-produced factors activate myeloid cells through TLR2 to stimulate metastasis. Nature.

[26] Rogers TL, Holen I (2011). Tumour macrophages as potential targets of bisphosphonates. J Transl Med.

[27] Erreni M, Mantovani A, Allavena P (2011). Tumor-associated macrophages (TAM) and inflammation in colorectal cancer. Cancer Microenviron.

[28] Sica A, Schioppa T, Mantovani A, Allavena P (2006). Tumour-associated macrophages are a distinct M2 polarised population promoting tumour progression: potential targets of anti-cancer therapy. Eur J Cancer.

[29] Barbera-Guillem E, Nyhus JK, Wolford CC, Friece CR, Sampsel JW (2002). Vascular endothelial growth factor secretion by tumor-infiltrating macrophages essentially supports tumor angiogenesis, and IgG immune complexes potentiate the process. Cancer Res.

[30] Pancione M, Forte N, Sabatino L (2009). Reduced beta-catenin and peroxisome proliferator-activated receptor-gamma expression levels are associated with colorectal cancer metastatic progression: correlation with tumor-associated macrophages, cyclooxygenase 2, and patient outcome. Hum Pathol.

[31] Bailey C, Negus R, Morris A (2007). Chemokine expression is associated with the accumulation of tumour associated macrophages (TAMs) and progression in human colorectal cancer. Clin Exp Metastasis.

[32] Algars A, Irjala H, Vaittinen S (2012). Type and location of tumor-infiltrating macrophages and lymphatic vessels predict survival of colorectal cancer patients. Int J Cancer.

[33] Forssell J, Oberg A, Henriksson ML, Stenling R, Jung A, Palmqvist R (2007). High macrophage infiltration along the tumor front correlates with improved survival in colon cancer. Clin Cancer Res.

[34] Zhou Q, Peng RQ, Wu XJ (2010). The density of macrophages in the invasive front is inversely correlated to liver metastasis in colon cancer. J Transl Med.

[35] Herrera M, Herrera A, Dominguez G (2013). Cancer-associated fibroblast and M2 macrophage markers together predict outcome in colorectal cancer patients. Cancer Sci.

[36] Recalcati S, Locati M, Marini A (2010). Differential regulation of iron homeostasis during human macrophage polarized activation. Eur J Immunol.

[37] Jedinak A, Dudhgaonkar S, Sliva D (2010). Activated macrophages induce metastatic behavior of colon cancer cells. Immunobiology.

[38] Park EK, Jung HS, Yang HI, Yoo MC, Kim C, Kim KS (2007). Optimized THP-1 differentiation is required for the detection of responses to weak stimuli. Inflamm Res.

[39] Lolmede K, Campana L, Vezzoli M (2009). Inflammatory and alternatively activated human macrophages attract vessel-associated stem cells, relying on separate HMGB1- and MMP-9-dependent pathways. J Leukoc Biol.

[40] Sica A, Larghi P, Mancino A (2008). Macrophage polarization in tumour progression. Semin Cancer Biol.

[41] Gordon S, Martinez FO (2010). Alternative activation of macrophages: mechanism and functions. Immunity.

[42] Kwan WH, Boix C, Gougelet N, Fridman WH, Mueller CG (2007). LPS induces rapid IL-10 release by M-CSF-conditioned tolerogenic dendritic cell precursors. J Leukoc Biol.

[43] Park ES, Yoo JM, Yoo HS, Yoon DY, Yun YP, Hong J (2012). IL-32gamma enhances TNF-alpha-induced cell death in colon cancer. Mol Carcinog.

[44] Min HY, Chung HJ, Kim EH, Kim S, Park EJ, Lee SK (2010). Inhibition of cell growth and potentiation of tumor necrosis factor-alpha (TNF-alpha)-induced apoptosis by a phenanthroindolizidine alkaloid antofine in human colon cancer cells. Biochem Pharmacol.

[45] Rivas MA, Carnevale RP, Proietti CJ (2008). TNF alpha acting on TNFR1 promotes breast cancer growth via p42/P44 MAPK, JNK, Akt and NF-kappa B-dependent pathways. Exp Cell Res.

[46] Gasperini S, Marchi M, Calzetti F (1999). Gene expression and production of the monokine induced by IFN-gamma (MIG), IFN-inducible T cell alpha chemoattractant (I-TAC), and IFN-gamma-inducible protein-10 (IP-10) chemokines by human neutrophils. J Immunol.

[47] Erreni M, Bianchi P, Laghi L (2009). Expression of chemokines and chemokine receptors in human colon cancer. Methods Enzymol.

[48] Han X, Wu Z, Di J (2011). CXCL9 attenuated chemo-therapy-induced intestinal mucositis by inhibiting proliferation and reducing apoptosis. Biomed Pharmacother.

[49] Andersson A, Srivastava MK, Harris-White M (2011). Role of CXCR3 ligands in IL-7/IL-7R alpha-Fc-mediated antitumor activity in lung cancer. Clin Cancer Res.

